# Association of Supply Type with Fecal Contamination of Source Water and Household Stored Drinking Water in Developing Countries: A Bivariate Meta-analysis

**DOI:** 10.1289/ehp.1409002

**Published:** 2015-05-08

**Authors:** Katherine F. Shields, Robert E.S. Bain, Ryan Cronk, Jim A. Wright, Jamie Bartram

**Affiliations:** 1 The Water Institute, University of North Carolina at Chapel Hill, Chapel Hill North Carolina, USA; 2 UNICEF, New York, New York, USA; 3 Geography and Environment, University of Southampton, Southampton, United Kingdom

## Abstract

**Background:**

Access to safe drinking water is essential for health. Monitoring access to drinking water focuses on water supply type at the source, but there is limited evidence on whether quality differences at the source persist in water stored in the household.

**Objectives:**

We assessed the extent of fecal contamination at the source and in household stored water (HSW) and explored the relationship between contamination at each sampling point and water supply type.

**Methods:**

We performed a bivariate random-effects meta-analysis of 45 studies, identified through a systematic review, that reported either the proportion of samples free of fecal indicator bacteria and/or individual sample bacteria counts for source and HSW, disaggregated by supply type.

**Results:**

Water quality deteriorated substantially between source and stored water. The mean percentage of contaminated samples (noncompliance) at the source was 46% (95% CI: 33, 60%), whereas mean noncompliance in HSW was 75% (95% CI: 64, 84%). Water supply type was significantly associated with noncompliance at the source (*p* < 0.001) and in HSW (*p* = 0.03). Source water (OR = 0.2; 95% CI: 0.1, 0.5) and HSW (OR = 0.3; 95% CI: 0.2, 0.8) from piped supplies had significantly lower odds of contamination compared with non-piped water, potentially due to residual chlorine.

**Conclusions:**

Piped water is less likely to be contaminated compared with other water supply types at both the source and in HSW. A focus on upgrading water services to piped supplies may help improve safety, including for those drinking stored water.

**Citation:**

Shields KF, Bain RE, Cronk R, Wright JA, Bartram J. 2015. Association of supply type with fecal contamination of source water and household stored drinking water in developing countries: a bivariate meta-analysis. Environ Health Perspect 123:1222–1231; http://dx.doi.org/10.1289/ehp.1409002

## Introduction

The health consequences of drinking fecally contaminated water, particularly for young children and immunocompromised individuals, have long been recognized (Gerba et al. 1996). International development initiatives, including the International Drinking Water Supply and Sanitation Decade in the 1980s and the more recent Millennium Development Goals (MDGs), have focused global policy attention on access to safe water (Bradley and Bartram 2013). Access to safe drinking water is monitored by the Joint Monitoring Programme for Water Supply and Sanitation (JMP) of the World Health Organization (WHO) and UNICEF (United Nations Children’s Fund) using the dichotomous indicator of the proportion of the population using an “improved” drinking water supply, which includes piped water, boreholes, protected springs and dug wells, and rainwater. Unprotected springs and dug wells, carts with small tanks, tanker trucks, and surface water are considered “unimproved” (Bartram et al. 2014). Although the WHO and UNICEF declared that the world had met the drinking water target in 2010, as assessed by use of this indicator, they cautioned that it is likely that the number of people using “safe” water had been overestimated (WHO/UNICEF 2012). Assessments that take source water quality into account suggest that between 1.8 and 3 billion people, or 28–47% of the global population, used unsafe water or water from sanitarily unsafe supplies in 2010 (Onda et al. 2012). Because many improved supplies are remote from households, they require transportation and storage of drinking water. Even when water is piped into a dwelling or yard, water storage may be required due to intermittent or unreliable supply.

Recognition of contamination during transport and household storage has sparked debate on the relative importance of water quality and treatment at the source versus point of use (PoU), the point at which drinking water is consumed (Boisson et al. 2013; Clasen and Cairncross 2004; Mintz et al. 2001; Trevett et al. 2004; VanDerslice and Briscoe 1993). Some have probed the health significance of intra- versus extra-household contamination. For example, VanDerslice and Briscoe (1993) suggested that most household members have immunity to pathogens already circulating in the household. Extra-household contamination poses a greater health risk, they argued, because it has the potential of bringing new pathogens into the household. Even if no immunity were present, transmission of pathogens via stored water would be inefficient relative to other transmission pathways. In contrast, Trevett et al. (2005) constructed a model for contamination of water stored in the household, theorizing that VanDerslice and Briscoe (1993) obtained their results because their study population had a low risk for contamination of stored water. Highly contaminated stored water would have a greater effect on health Trevett et al. argued. Others have explored the treatment options, suggesting that household treatment is essential, at least for the present, because source water treatment is more time and resource intensive (Mintz et al. 2001). Finally, Clasen and Bastable (2003) and Wright et al. (2004) emphasized the need to measure and monitor quality at the PoU in addition to the source.

In their review, Wright et al. (2004) found that, in half of the studies included, contamination was greater at the PoU than at the source, and in no case did microbiological water quality improve significantly from the source to the PoU. Wright et al. (2004) were unable to explore differences between types of water supplies; in their analysis, unprotected well water and household connections delivering chlorinated water were treated as equal. Thus, our goal here is to build upon the work of Wright et al. (2004) by exploring contamination at the water source separately from contamination of household stored water (HSW), and examining whether factors such as water provenance, rural/urban setting, and indicator organism have a differential effect on contamination at each location. In light of the findings of Bain et al. (2014b) that water supply type is strongly associated with noncompliance [i.e., the percentage of water samples contaminated by fecal indicator bacteria (FIB)], we describe the supply types in as much detail as possible instead of aggregating them into improved and unimproved categories only. We use “water source” to mean the point of collection or receipt by the household. This includes water as taken from a river, a handpump, community tap stand, or tap in a household, as well as water as it is received from a vendor or tanker truck. Here, we use the term “HSW” to refer to any water stored in the home, rather than water at the PoU, referring to the point that water is drawn from immediately prior to consumption or use. The distinction is important because water may be transferred to another container, and it is unclear how long water is stored prior to consumption by household members.

## Methods

*Study selection*. Studies used in this review and meta-analysis are a subset of those included in the systematic review by Bain et al. (2014b) who analyzed 319 studies based on the inclusion criteria that studies *a*) reported on water quality at the source or in HSW, with sources not classified as surface water by the JMP; and *b*) included extractable data on thermotolerant coliforms (TTC) or *Escherichia coli,* with sample volumes of ≥ 10 mL and at least 10 samples from different water supplies of a given type. Articles in English, Spanish, French, and Portuguese that described studies in developing countries, as defined by the MDG regions, and published between January 1990 and August 2013 were included.

We screened abstracts of the 319 articles analyzed by Bain et al. (2014b) to identify those reporting data on water quality at both the source and in HSW. The most common definition of HSW was water sampled from a household storage container. Some studies provided additional detail of container type (e.g., household water jug, opaque containers, bucket) and storage duration (e.g., ≤ 24 hr). A few studies referred simply to household water, with storage implied in the sampling description. Where abstracts were unclear, methods sections were reviewed. Full texts were reviewed to identify articles that reported sampling both water sources and HSW. If methods sections indicated that water quality data at both the source and in HSW were collected, but either data were not reported from both sampling points, or reported data were not disaggregated by water supply type, authors were contacted at least twice to request data. Studies were excluded if they did not report data about both source and stored water, if the data were not disaggregated by supply type, or if < 10 samples were taken at either the source or from HSW. Rainwater collection, which acts as both a source and HSW, was excluded.

Some studies sampled pairs, taking one water sample from stored water in a household and one sample from the water source used by the household. Other studies sampled a number of HSW and all available sources, creating pairs *post hoc* by linking stored water data of a particular household to source water data based on reported supply type.

Study information extracted by Bain et al. (2014b) and used in the present analysis included *a*) supply type (unprotected well, unprotected spring, unspecified well or spring, protected well, protected spring, borehole, piped, surface, tanker truck, or bottled water); *b*) source treatment (reported chlorination and assessed residual chlorine) and household water treatment (HWT) (boiling, filtration, chlorination); *c*) compliance (percentage of samples ≥ 10 mL that were free of *E. coli* or TTC); *d*) mean, geometric mean, and/or median level of contamination by water supply type; *e*) number of samples collected at source and from stored water; *f* ) location (urban or rural as defined by the authors, ‘‘mixed’ if the setting was mixed); *g*) study country; *h*) year of publication; *i*) study design; and *j*) study quality information as described by Bain et al. (2014b) (see Supplemental Material, Table S1). For each item in Supplemental Material, Table S1, studies were assigned a point, resulting in a study quality score of between 0 and 13; studies were then separated into terciles of “low”(< 7), “medium” (7–9), and “high” (> 9) quality. World Bank classifications from 2013 were used to determine country income levels (World Bank 2013). For intervention studies, baseline water quality data for both the intervention and control groups were used where possible. If baseline data were not available and for case–control studies, water quality data from the control group were used.

*Meta-analysis*. Meta-analysis was used to explore factors that were associated with differences in water quality, including water supply characteristics, study setting, study characteristics, and reporting format. We used bivariate random effects meta-analysis and meta-regression to analyze noncompliance at the source, noncompliance of HSW, and the odds ratio (OR) between the two simultaneously. Studies in which water was sampled from the drinking cup rather than from household storage, studies in which all sampled HSW was known to have been treated in the household, and studies that reported only central tendency of FIB were excluded from the meta-analysis.

Analysis was completed using PROC GLIMMIX in SAS 9.4 (SAS Institute Inc.). Number of “events” (water samples contaminated at a given sampling point) compared with “trials” (total number of samples analyzed at a given sampling point) were modeled using a bivariate distribution and a logit link function. Degrees of freedom were set at 1,000 in order to produce *z*-statistics rather than a Student *t*-statistic (Reitsma et al. 2005). Covariates were analyzed as interactions with each sampling point (source and HSW) to avoid assuming a covariate had the same effect at both.

To avoid assuming a fixed relationship between quality at the source and quality of HSW for any given water supply, we used the water sampling point (source or HSW) to model random effects. Pooled logit estimates at both sampling points were back-transformed to report mean noncompliance on the scale of the original data. Overall significance levels for each covariate were calculated using Type III tests of the fixed effects of the interaction between sampling point (source or HSW) and the covariate of interest. Odds ratios were calculated to compare levels of categorical variables (e.g., improved vs. unimproved water supplies) at both the source and in HSW and to compare noncompliance between source and HSW, after adjustment for water supply type. The 95% confidence regions for mean noncompliance were calculated using the geometric relationships between variance and correlation (Pakula 2008) and plotted using SAS 9.4.

We used the multivariate *I_R_*^2^ statistic developed by Jackson et al. (2012) (as described in Zhou and Dendukuri 2014) to quantify study heterogeneity. To examine publication bias, we calculated log odds ratios between HSW and source, with log odds > 0 indicating higher noncompliance in HSW. A funnel plot was created, and the trim and fill method applied using STATA 12 (StataCorp).

## Results

**Figure 1:**
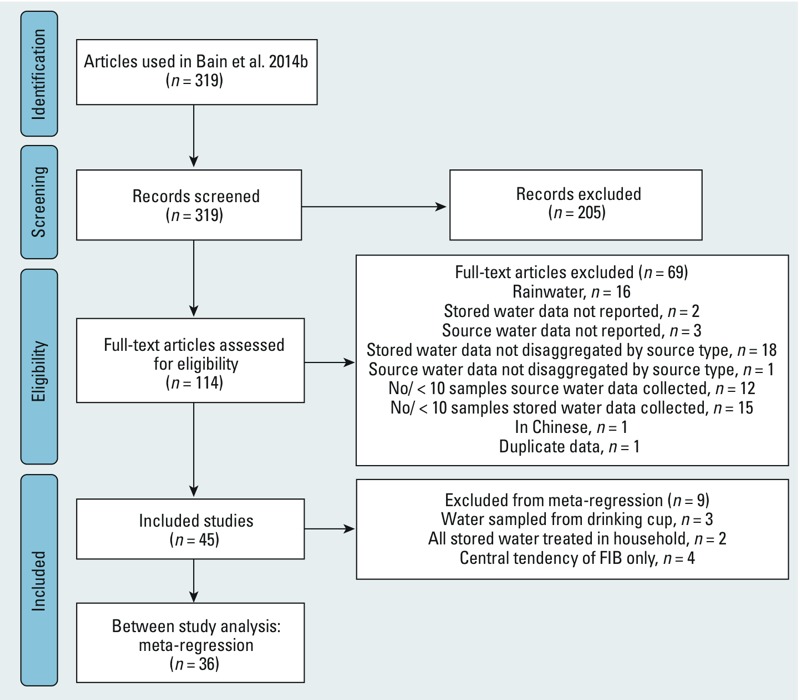
Selection of articles for meta-analysis.

*Study characteristics*. A total of 319 abstracts were screened and 114 full text articles assessed (Figure 1).

Characteristics of the 45 included studies are summarized in Table 1. Descriptive statistics for included studies are presented in Table 2. Most studies were cross-sectional (*n* = 27, 60%), whereas a quarter were intervention studies (*n* = 12, 27%). Seven of the MDG regions were represented in this review (see Supplemental Material, Figure S1), with most studies taking place in sub-Saharan Africa (*n* = 24, 53%), 10 in South Asia (22%), and 7 cross-sectional in Latin America and the Caribbean (16%). Low- and lower-middle-income countries dominated (*n* = 21 and 17, respectively). A similar number of studies had rural and urban settings (*n* = 20 and 21, respectively), with 4 studies having mixed settings. The majority of studies used *E. coli* (*n* = 25, 56%) rather than TTC as the fecal indicator bacteria.

**Table 1:** Included studies.

Study	MDG region	Country income level	Setting	Indicator	Study design	Study quality	Random selection	Publication year	Measure of central tendency
Abdellah et al. 2012	Sub-Saharan Africa	Low	Rural	EC	Cross-sectional	Low	No	After 2009	Yes
Agard et al. 2002	Latin America and the Caribbean	High	Urban	EC	Cross-sectional	Medium	Yes	In or before 2009	No
Aldana 2010	Latin America and the Caribbean	Lower-middle	Both	TTC	Cross-sectional	High	Yes	After 2009	No
Aliev et al. 2010	Caucuses and Central Asia	Low	Both	TTC	Cross-sectional	High	Yes	After 2009	Yes
Austin 1994	Sub-Saharan Africa	Low	Rural	TTC	Intervention	Low	No	In or before 2009	Yes
Baker et al. 2013	Sub-Saharan Africa	Low	Urban	EC	Cross-sectional	High	Yes	After 2009	Yes
Chemuliti et al. 2002	Sub-Saharan Africa	Low	Urban	TTC	Cross-sectional	Medium	No	In or before 2009	Yes
Chung 2011	Sub-Saharan Africa	Low	Both	EC	Cross-sectional	Low	No	After 2009	Yes
Cronin et al. 2006	Sub-Saharan Africa	Low	Urban	TTC	Longitudinal	Low	No	In or before 2009	Yes
de Sá et al. 2005	Latin America and the Caribbean	Upper-middle	Urban	TTC	Longitudinal	High	Yes	In or before 2009	Yes
Elala et al. 2011	South Asia	Lower-middle	Urban	TTC	Cross-sectional	Medium	No	After 2009	No
Eshcol et al. 2009	South Asia	Lower-middle	Urban	EC	Cross-sectional	Medium	No	In or before 2009	Yes
Fiore et al. 2010^*a*^**	Latin America and the Caribbean	Lower-middle	Rural	EC	Intervention	Low	No	After 2009	No
Firth et al. 2010	South Asia	Lower-middle	Rural	TTC	Intervention	Low	No	After 2009	Yes
Genthe et al. 1997	Sub-Saharan Africa	Upper-middle	Urban	EC	Case–control	Low	No	In or before 2009	Yes
Handzel 1998	South Asia	Low	Urban	EC	Intervention	High	Yes	In or before 2009	Yes
Holm 2012^*b*^**	Sub-Saharan Africa	Low	Urban	EC	Cross-sectional	Medium	No	After 2009	Yes
Hoque et al. 2006	South Asia	Low	Rural	TTC	Cross-sectional	High	No	In or before 2009	Yes
Jagals et al 2013	Sub-Saharan Africa	Upper-middle	Rural	EC	Cross-sectional	Low	No	After 2009	No
Jagals et al. 1999	Sub-Saharan Africa	Upper-middle	Urban	TTC	Intervention	Medium	No	In or before 2009	Yes
Jagals et al. 1997	Sub-Saharan Africa	Upper-middle	Urban	TTC	Cross-sectional	Medium	Yes	In or before 2009	Yes
Kanyerere et al. 2012	Sub-Saharan Africa	Low	Rural	EC	Cross-sectional	Medium	Yes	After 2009	Yes
Khush et al. 2009	South Asia	Lower-middle	Rural	EC	Intervention	Low	No	In or before 2009	Yes
Klasen et al. 2012^*b*^**	Western Asia	Lower-middle	Urban	EC	Intervention	Low	Yes	After 2009	Yes
Kremer et al. 2011	Sub-Saharan Africa	Low	Rural	EC	Intervention	Medium	Yes	After 2009	Yes
Kumpel and Nelson 2013^*b*^**	South Asia	Lower-middle	Urban	EC	Longitudinal	Medium	No	After 2009	Yes
Lacey et al. 2011	Latin America and the Caribbean	Lower-middle	Rural	EC	Cross-sectional	High	Yes	After 2009	Yes
Magrath 2006	Sub-Saharan Africa	Low	Rural	TTC	Cross-sectional	Low	No	In or before 2009	Yes
Mazengia et al. 2002	Sub-Saharan Africa	Low	Rural	TTC	Intervention	Medium	No	In or before 2009	Yes
Mertens et al. 1990	South Asia	Lower-middle	Rural	TTC	Case–control	High	No	In or before 2009	Yes
Oloruntoba and Sridhar 2007	Sub-Saharan Africa	Lower-middle	Urban	EC	Cross-sectional	High	Yes	In or before 2009	Yes
Pickering et al. 2010	Sub-Saharan Africa	Low	Urban	EC	Cross-sectional	High	Yes	After 2009	Yes
Platenburg and Zaki 1993	North Africa	Lower-middle	Rural	TTC	Intervention	Low	No	In or before 2009	Yes
Potgieter et al. 2009^*b*^**	Sub-Saharan Africa	Upper-middle	Rural	EC	Intervention	Medium	Yes	In or before 2009	Yes
Quick et al. 2002	Sub-Saharan Africa	Lower-middle	Urban	EC	Intervention	High	Yes	In or before 2009	No
Rosa et al. 2010^*a*^**	Latin America and the Caribbean	Lower-middle	Rural	TTC	Cross-sectional	Medium	No	After 2009	Yes
Shaheed et al. 2014	South East Asia	Low	Urban	EC	Cross-sectional	Medium	Yes	After 2009	Yes
Shar et al. 2010	South Asia	Lower-middle	Urban	EC	Cross-sectional	Low	No	After 2009	Yes
Shrestha et al. 2013	South Asia	Low	Rural	EC	Cross-sectional	Low	No	After 2009	No
Simango et al. 1992	Sub-Saharan Africa	Low	Rural	EC	Cross-sectional	Medium	No	In or before 2009	No
Stoler et al. 2012	Sub-Saharan Africa	Lower-middle	Urban	EC	Cross-sectional	Low	No	After 2009	Yes
Sutton et al. 2012	Sub-Saharan Africa	Low	Rural	TTC	Cross-sectional	Low	No	After 2009	Yes
Tabor et al. 2011	Sub-Saharan Africa	Low	Urban	TTC	Cross-sectional	Medium	Yes	After 2009	No
Tadesse et al. 2010	Sub-Saharan Africa	Low	Both	TTC	Cross-sectional	High	No	After 2009	Yes
Trevett et al. 2004	Latin America and the Caribbean	Lower-middle	Rural	TTC	Longitudinal	Medium	No	In or before 2009	Yes
Abbreviations: EC, *Escherichia coli*; MDG, Millennium Development Goal; TTC, thermotolerant coliforms. ^***a***^Study excluded from meta-regression because all samples were reportedly treated in the household. ^***b***^Study excluded from meta-regression because water was sampled from the drinking cup rather from the household water storage container.									

**Table 2:** Characteristics of included studies.

Variable	Studies *n* (%)	Water supply types evaluated *n* (%)	Source water samples *n* (%)	Household stored water samples *n* (%)
Total	45	65	10,934	12,523
MDG region
Caucuses and Central Asia	1 (2)	2 (3)	1,319 (12)	119 (1)
Latin America and the Caribbean	7 (16)	10 (15)	2,247 (21)	1,301 (10)
North Africa	1 (2)	1 (2)	189 (2)	183 (1)
South Asia	10 (22)	13 (20)	2,703 (25)	5,904 (47)
South East Asia	1 (2)	1 (2)	124 (1)	124 (1)
Sub-Saharan Africa	24 (53)	36 (55)	3,854 (35)	4,391 (35)
Western Asia	1 (2)	2 (3)	498 (5)	501 (4)
Country income level
High	1 (2)	1 (2)	81 (1)	104 (1)
Upper-middle	6 (13)	7 (11)	504 (5)	866 (7)
Lower-middle	17 (38)	25 (38)	5,288 (48)	7,468 (60)
Low	21 (47)	32 (49)	5,061 (46)	4,085 (33)
Setting
Rural	20 (44)	27 (42)	3,466 (32)	8,181 (65)
Urban	21 (47)	28 (43)	3,035 (28)	3,871 (31)
Both	4 (9)	10 (15)	4,433 (41)	471 (4)
FIB
*E. coli*	25 (56)	31 (48)	3,659 (33)	8,177 (65)
TTC	20 (44)	34 (52)	7,275 (67)	4,346 (35)
Study design
Case–control	2 (4)	4 (6)	1,068 (10)	1,687 (13)
Cross-sectional	27 (60)	40 (62)	6,482 (59)	3,293 (26)
Intervention	12 (27)	14 (22)	2,286 (21)	6,123 (49)
Longitudinal	4 (9)	7 (11)	1,098 (10)	1,420 (11)
Study quality
Low	16 (36)	23 (35)	2,440 (22)	4,831 (39)
Medium	17 (38)	18 (28)	1,931 (18)	3,821 (31)
High	12 (27)	24 (37)	6,563 (60)	3,871 (31)
Random selection^*a*^
No	28 (62)	37 (57)	4,880 (45)	8,109 (65)
Yes	17 (38)	28 (43)	6,054 (55)	4,414 (35)
Publication year
In or before 2009	21 (47)	30 (46)	3,887 (36)	7,278 (58)
After 2009	24 (53)	35 (54)	7,047 (64)	5,245 (42)
Measure of central tendency^*b*^
No	9 (20)	12 (18)	1,985 (18)	790 (6)
Yes	36 (80)	53 (82)	8,949 (82)	11,733 (94)
Water supply type
Improved
Piped		31 (48)	5,425 (50)	6,316 (50)
Borehole		12 (18)	1,749 (16)	1,736 (14)
Protected well		8 (12)	1,867 (17)	1,724 (14)
Protected spring		2 (3)	654 (6)	59 (0)
Unimproved
Unprotected well		4 (6)	316 (3)	220 (2)
Unprotected spring		1 (2)	193 (2)	1,445 (12)
Tanker truck		1 (2)	211 (2)	212 (2)
Unspecified
Unspecified well		6 (9)	519 (5)	811 (6)
Treatment of piped sources^*c*^
Reported chlorination; residual chlorine assessed		8 (26)	1,416 (26)	937 (15)
Reported chlorination; residual chlorine not assessed		6 (19)	578 (11)	943 (15)
Inconsistent chlorination; residual chlorine not assessed		6 (19)	2,026 (37)	407 (6)
Not treated		2 (6)	240 (4)	216 (3)
Not reported		9 (29)	1,165 (21)	3,813 (60)
Abbreviations: FIB, fecal indicator bacteria; MDG, Millennium Development Goal; TTC, thermotolerant coliforms. ^***a***^Random selection was assessed through the question “Was sampling randomized over a given study area or population?” ^***b***^Studies were considered to include a measure of central tendency if they reported mean, geometric mean, and/or median level of contamination by water supply type. ^***c***^The total *n* for this set of characteristics is 31, corresponding to the number of studies that reported information on piped supplies. No other supply types were reported to be chlorinated or to have been tested for residual chlorine.				

The 45 included studies reported, on average, 1.5 water supply types for a total of 65 water supply observations, with 10,934 total water samples taken at the source and 12,523 samples from HSW (Table 2). Thirty-two studies included source and HSW comparisons for only one supply type, whereas 6 studies included two supply types, 6 studies included three supply types and 1 study included four supply types. Nearly half of the water supply observations were piped (*n* = 31, 49%); one third were “other improved supplies” (*n* = 22), including boreholes, protected wells, and protected springs. Six supplies were classified as unimproved (9%). In 6 cases, the protection status of wells could not be determined, and these wells were treated as a separate supply category.

Of the 31 studies reporting on piped supplies, about half were reported to be chlorinated (*n* = 14); for an additional 6 supplies, authors noted inconsistent or irregular chlorination (Table 2). Two supplies were not disinfected, and for the remaining 9, chlorination status was not reported. All of the studies reporting inconsistent or irregular chlorination (*n* = 6) assessed residual chlorine at the source, in HSW, or both. However, in only 8 of the 14 supplies reported as chlorinated was residual chlorine detected. No other supply types were reported to be chlorinated. Residual chlorine was variously reported as an average or range of chlorine concentration or proportion of samples containing > 0.5 or 0.2 mg/L, thus preventing its inclusion in meta-analysis.

Two studies (Fiore et al. 2010; Rosa et al. 2010) reported only on HSW treated in the home. Another study (Mertens et al. 1990) analyzed one boiled and one unboiled stored water sample from each household. We excluded these boiled water data because pairs of stored water samples from the same household are unlikely to be statistically independent. Although many of the other studies mentioned household water treatment and storage practices, there was a lack of comparable data across the studies.

*Between-study analysis*. Using a bivariate random-effects model, water quality was found to be significantly worse in HSW compared with the source. Mean noncompliance at the source was found to be 46% (95% CI: 33, 60%), with mean noncompliance in HSW at 75% (95% CI: 64, 84%) (Table 3), an unadjusted odds ratio of 3.5 (2.5, 5.0) (Table 5). Noncompliance at the source (*p* < 0.001) and in HSW (*p* = 0.03) was significantly associated with water supply type. At the source, mean noncompliance in piped water was 25% (95% CI: 15, 40%). In HSW, mean noncompliance for piped water was higher than at the source at 62% (95% CI: 44, 77%) (Table 3). Unprotected and unspecified wells had the highest mean noncompliance at both the source and in HSW. Protected and unprotected springs also had low rates of noncompliance at both the source and in the household; however, the confidence intervals (CIs) around these estimates were wide. After adjusting for supply type, HSW had 2.3 higher odds of contamination than source water (95% CI: 1.4, 3.9) (Table 5).

**Table 3:** Mean proportion of noncompliant samples from between studies meta-regression.

Covariate	Source			HSW		
	*n* Water samples	Mean noncompliance (95% CI)	*p*‑Value	*n* Water samples	Mean noncompliance (95% CI)	*p*‑Value
Unadjusted	9,198	46 (33, 60)	0.59	10,557	75 (64, 84)	< 0.001
Water supply type			< 0.001			0.03
Improved
Piped	4,195	25 (15, 40)		4,801	62 (44, 77)
Borehole	1,749	43 (22, 66)		1,736	83 (63, 93)
Protected well	1,753	70 (41, 89)		1,666	80 (51, 94)
Protected spring	654	35 (5, 84)		59	46 (6, 91)
Unimproved
Unprotected well	286	94 (69, 99)		190	94 (65, 99)
Unprotected spring	654	5 (0, 61)		1,445	14 (0, 83)
Unspecified
Unspecified well	368	91 (68, 98)		660	94 (77, 99)
MDG region			0.91			0.17
Caucuses and Central Asia	1,319	14 (1, 78)		119	10 (1, 61)
Latin America and the Caribbean	1,872	54 (20, 84)		944	67 (36, 88)
North Africa	189	61 (2, 99)		183	89 (17, 100)
South Asia	2,166	51 (22, 79)		5,308	85 (65, 94)
South East Asia	124	53 (1, 99)		124	82 (10, 99)
Sub-Saharan Africa	3,528	44 (26, 63)		5,308	76 (61, 86)
Country income level			0.03			0.23
High	81	33 (1, 96)		104	67 (5, 99)
Upper-middle	322	9 (1, 43)		442	39 (9, 81)
Lower-middle	3,878	68 (47, 84)		6,014	84 (68, 92)
Low	4,917	39 (23, 56)		3,997	73 (57, 84)
Setting			0.44			0.67
Rural	2,961	55 (32, 77)		7,756	79 (62, 89)
Urban	1,804	42 (21, 66)		2,330	83 (67, 92)
FIB			0.03			0.42
*E. coli*	2,427	30 (16, 50)		6,889	71 (52, 84)
TTC	6,771	59 (41, 75)		3,668	79 (65, 88)
Study design			0.17			0.21
Case–control	1,068	52 (12, 89)		1,687	70 (26, 94)
Cross-sectional	6,199	72 (37, 92)		2,957	91 (72, 98)
Intervention	1,370	69 (30, 92)		5,089	84 (52, 96)
Longitudinal	591	36 (22, 52)		824	69 (54, 81)
Study quality			0.44			0.66
Low	1,791	44 (24, 67)		4,179	70 (49, 85)
Medium	844	31 (12, 62)		2,507	72 (45, 89)
High	6,563	55 (34, 74)		3,871	80 (64, 90)
Random selection			0.61			0.99
No	3,687	49 (31, 68)		6,754	75 (60, 86)
Yes	5,511	42 (24, 63)		3,803	75 (58, 87)
Publication year			0.01			0.007
In or before 2009	3,591	64 (45, 79)		6,796	86 (75, 93)
After 2009	5,607	31 (18, 48)		3,761	61 (45, 76)
Measure of central tendency			0.54			0.30
No	1,834	38 (15, 68)		639	64 (36, 85)
Yes	7,364	48 (33, 64)		9,918	78 (66, 86)
Data were derived from bivariate random-effects regression of the number of noncompliant samples out of the total number of samples. The unadjusted model contained fixed effects of source noncompliance and HSW noncompliance. All adjusted models included one covariate of interest an interaction with source noncompliance and an interaction term with HSW noncompliance. Model estimates were back-transformed to derive estimates of mean compliance on the scale of the original data. *p*-Values were calculated using the type III test of fixed effects.						

**Table 4:** Odds ratios for microbial noncompliance, comparing source, study setting, and study design characteristics calculated for source and household stored water samples.

Contrast	Source		HSW	
	OR (95% CI)	*p*-Value	OR (95% CI)	*p*-Value
Unimproved vs. improved supplies	6.5 (1.0, 43.9)	0.06	2.0 (0.3, 14.4)	0.48
Improved supplies: piped vs. other	0.3 (0.1, 0.8)	0.01	0.4 (0.2, 1.2)	0.12
All supplies: piped vs. other	0.2 (0.1, 0.5)	0.002	0.3 (0.2, 0.8)	0.03
Unprotected vs. protected groundwater	3.5 (0.7, 18.5)	0.14	1.2 (0.2, 6.1)	0.84
Other MDG regions vs. sub-Saharan Africa	1.4 (0.4, 4.8)	0.59	1.2 (0.4, 3.4)	0.75
Longitudinal vs. cross-sectional	3.9 (0.8, 19.4)	0.09	2.4 (0.6, 9.8)	0.24
High quality vs. low quality	1.5 (0.4, 5.5)	0.51	1.7 (0.5, 5.5)	0.38
Published in or before 2009 vs. after 2009	4.0 (1.4, 11.6)	0.01	3.9 (1.5, 10.4)	0.007
Non-random selection vs. random selection	3.9 (0.9, 17.7)	0.07	3.9 (0.7, 21.7)	0.12
FIB: TTC vs. *E. coli*	1.3 (0.4, 4.2)	0.61	1.0 (0.4, 2.9)	0.99
Data were derived from bivariate random-effects regression of the number of noncompliant samples out of the total number of samples. All adjusted models included one covariate of interest, an interaction with source noncompliance, and an interaction term with HSW noncompliance. All levels of a variable were included in the models; however, if a variable had more than two levels, only two were selected to calculate an odds ratio.				

**Table 5:** Odds ratios for comparison of percentage of source and HSW noncompliance.

Contrast	OR (95% CI)	*p*-Value
Source vs. HSW (unadjusted)	3.5 (2.5, 5.0)	< 0.001
Source vs. HSW (adjusted^*a*^)	2.3 (1.4, 3.9)	0.001
^***a***^Adjusted for water supply type using the categorization from Table 2.		

**Figure 2:**
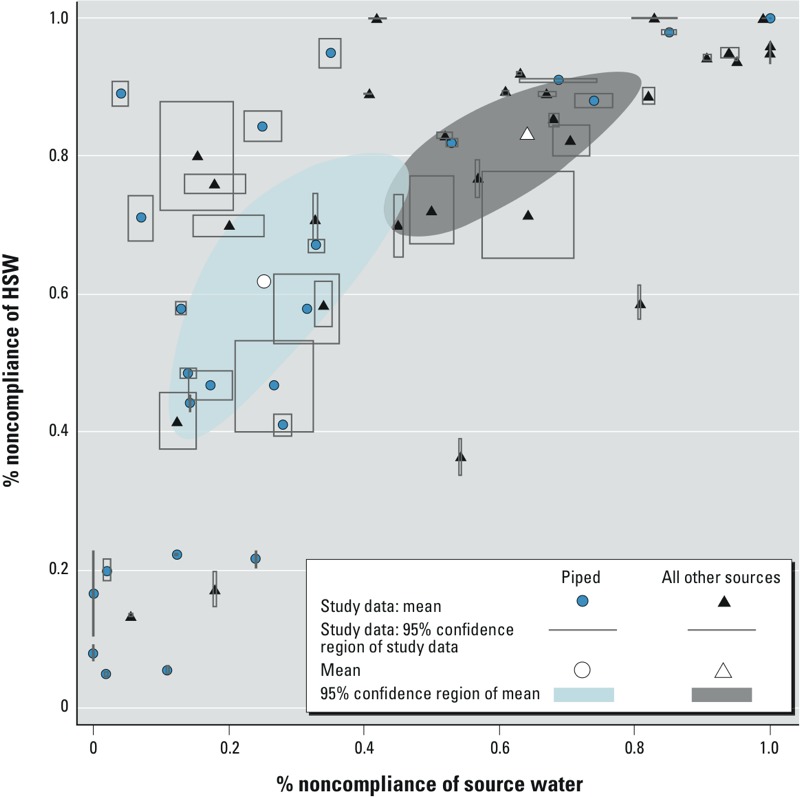
Percent noncompliance of source water versus household stored water (HSW) for the studies included in bivariate meta-regression together with meta-regression results. The filled symbols indicate noncompliance for the 55 water supplies from 36 studies; piped and all other sources of drinking water in a given study are treated separately. Gray lines indicate the 95% CIs of the study data, calculated using the standard formula for standard error of a proportion. The unfilled symbols indicate mean noncompliance of source and HSW, calculated using a bivariate random-effects model with dichotomous source type interacted with both source noncompliance and HSW noncompliance. The 95% confidence regions for mean noncompliance were calculated using the geometric relationships between variance and correlation (Pakula 2008). Model estimates and 95% confidence regions from meta-regression were back-transformed to derive estimates of mean compliance and 95% confidence regions on the scale of the original study data; the 95% confidence regions took the form of a rotated ellipse prior to back-transformation.

Piped supplies had lower odds of being contaminated than other improved supplies (OR = 0.3; 95% CI: 0.1, 0.8) and all other supply types (OR = 0.2; 95% CI: 0.1, 0.5) at the source (Table 4). HSW from piped supplies had significantly lower odds of contamination compared with all other supply types (OR = 0.3; 95% CI: 0.2, 0.8). Although the ellipses showing confidence limits for piped water versus water from other supplies overlap in Figure 2, the bivariate meta-regression indicated that odds ratios for noncompliant piped HSW versus source water was significantly different from the same odds ratio as calculated for other supply types (Table 4). The confidence limits overlap due to the correlation between noncompliance at the source and in HSW.

Country income level (*p* = 0.03) was significantly associated with water quality at the source when all supply types are aggregated (Table 3). Lower-middle income countries had the highest mean noncompliance at 68% (95% CI: 47, 84%).

The FIB used by a study was significantly associated with noncompliance at the source (*p* = 0.03), with mean noncompliance of TTC samples at 59% (95% CI: 41, 75%) and mean noncompliance of *E. coli* samples at 30% (95% CI: 16, 50%). There was a nonsignificant increase in the odds of source water noncompliance in longitudinal studies compared with cross-sectional studies (OR = 3.9; 95% CI: 0.8, 19.4), but the estimate was based on only four longitudinal studies (Table 3).

Studies published after 2009 (median year of included studies) had significantly lower mean noncompliance rates compared with studies published in or before 2009 for both the source (*p* = 0.01) and HSW (*p* = 0.007) when all supply types are aggregated. Samples taken at the source were 4.0 (95% CI: 1.4, 11.6) times more likely to be noncompliant and samples taken from HSW were 3.9 times (95% CI: 1.5, 10.4) more likely to be noncompliant for studies published in or before 2009 compared with studies published after 2009.

**Figure 3:**
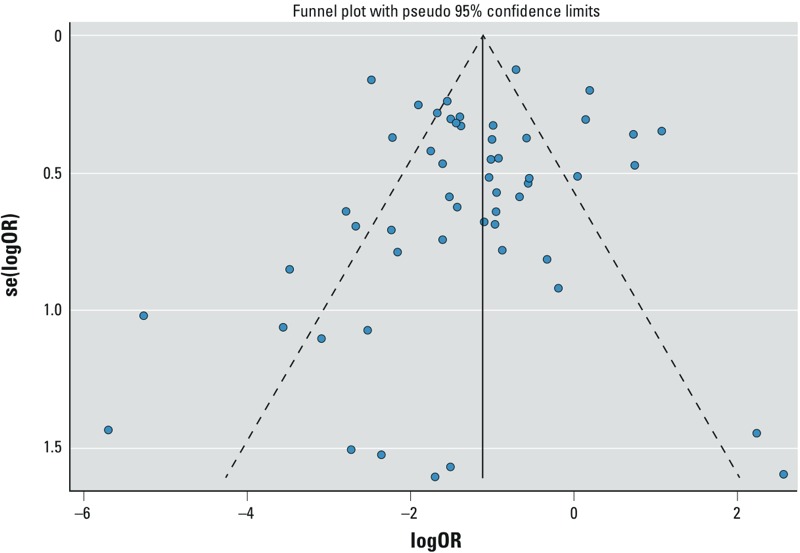
Funnel plot of log odds ratios of HSW versus source water. Log odds > 0 indicates that noncompliance is greater in HSW.

The heterogeneity of the studies was very high with a multivariate *I_R_*^2^ value of 0.91, indicating that 91% of total variance could be accounted for by between-study variance. There was no evidence of publication bias; the trim and fill test indicated no studies would need to be trimmed to create a symmetrical funnel plot (Figure 3).

## Discussion

Although the JMP estimated that 748 million people used unimproved water in 2012 (WHO/UNICEF 2014), several studies have modeled the global population drinking unsafe water through incorporation of water quality data at the source (Bain et al. 2014a; Onda et al. 2012). These refined estimates indicate that approximately 1.8 billion people lack access to safe drinking water (Onda et al. 2012), with 1.1 billion of these people using source water that is at least “moderate” risk (> 10 *E. coli* or TTC per 100 mL) (Bain et al. 2014a). Onda et al. (2012) further corrected these estimates for sanitary inspection scores of water sources, concluding that a further 1.2 billion people use water from sources with multiple sanitary risks. Because we have found that HSW is substantially more likely to be contaminated than water at the source, we suggest that even these refined estimates of the global population exposed to fecal contamination are likely to be underestimates.

Developed initially for its 2008 report, the JMP water ladder refined the concepts of “improved” and “unimproved” supplies (WHO/UNICEF 2008). It includes four rungs, descending from water piped on premises to other improved supplies, unimproved supplies (excluding surface water), and surface water. We found that source water from piped supplies was of significantly higher quality than that from other sources, and this held true for HSW also, providing evidence to support the water ladder and promotion of piped water.

The point of collection for piped supplies—community standpipes, piped on plot, and piped into the dwelling—is critical. The need for water storage is thought to be associated with distance to the collection point and reliability of the water source (Bain et al. 2014a). Although the JMP water ladder distinguishes between water piped on premises and other improved supplies, which include community standpipes, it was not possible to determine the location of the point of collection for many of the studies included in the review.

Piped water may be continuous, 24 hr/day 7 days/week, predictably intermittent, or unreliable. Some studies included in this review identified contamination related to noncontinuous flow, including bacterial growth (Agard et al. 2002; Kumpel and Nelson 2013). Others noted the need to store piped water if the supply is intermittent (Shaheed et al. 2014) which, as shown in our meta-analysis, may increase noncompliance. Finally, in our meta-analysis residual chlorine was found only for piped supplies (Table 2); however, we were unable to analyze the effects of residual chlorine on water quality due to diverse reporting methods. To enable such an investigation, we recommend that researchers report the presence of residual chlorine in drinking water samples tested for *E. coli*, especially those from piped supplies, using the WHO guideline values of 0.2 and 0.5 ppm (WHO 2011).

In our meta-analysis, we assessed the prevalence of noncompliant samples contaminated with *E. coli* or TTC, but not the public health impact of noncompliance. Our findings follow from Wright et al. (2004) as, in general, noncompliance is higher in HSW than at the source. However, we found that this relationship is modified by water supply type, with piped supplies having significantly lower odds of contamination at the source and in HSW than non-piped supplies. This finding may largely reflect the presence of residual chlorine in piped supplies that is uncommon among other supply types. Ensuring correct, consistent, and continued usage of water treatment by households using non-piped supplies has proven to be challenging (Brown and Clasen 2012) and an area of active research (Ahuja et al 2010). Although we anticipate that health impact will depend on different pathogens present in source and stored water, we argue that piped supplies may be safer because they are likely to have fewer pathogens from both. We suggest that “leapfrogging” households up the water ladder from unimproved sources to piped water could bring substantive health benefits. In addition, “leapfrogging” households could decrease abandoned investments, estimated at US$78 billion, which result from households passing stepwise through each rung of the water ladder, progressively abandoning their previous sources (Bain et al. 2013).

Leapfrogging households up the water ladder is unlikely to eliminate the need for water storage. Piped supplies that are unreliable or intermittent are common and necessitate water storage. It is widely believed that once households have predictable and reliable piped water on premises, storage behaviors will decline. However, Onda (2014) documented continued storage behavior, the reasons for which are poorly understood but may include anticipation of supply cuts (Onda 2014), taking advantage of the cooling activity of clay vessels (Klasen et al. 2012) or a refrigerator, convenience when a tap is on premises but not in the area for eating or drinking, and habit. To reduce storage practices, which may lead to higher noncompliance, a better understanding of the prevalence of and reasons for household water storage is necessary.

Contamination in HSW is widespread, and it therefore appears credible that household water treatment and safe storage (HWTS) may have an interim role, especially in rural areas where access to piped water is less common in all regions. We were unable to assess the impact of HWTS on HSW quality due to lack of disaggregation in most studies. One study included samples of untreated and reportedly boiled water stored in the household (Mertens et al. 1990). Reportedly boiled HSW had 36–52% lower noncompliance depending on supply type than did untreated HSW. Trevett et al. (2004), who found that well water noncompliance was lower in HSW, suggested that this apparent anomaly could have been due to inadvertent HSW treatment, such as dipping a ladle cleaned with bleach into the water. In general, however, both the efficacy of some HWTS methods and the determinants of consistent proper usage of HWTS remain inadequately understood; thus, the actual health benefits of HWTS remain unclear (Boisson et al. 2013; Brown and Clasen 2012; Enger et al. 2012).

One of the primary modes of contamination of HSW is contact with dirty hands and utensils (Psutka et al. 2011). Because of interaction with household hygiene, which lies in the private domain, stored water quality may not fall neatly into regulatory frameworks in the same manner as source water quality. The health sector should therefore play a key role in surveillance and policy making to address the interaction between stored water quality and household hygiene (Rehfuess et al. 2009).

*Methodological challenges: outcome level*. The dichotomous measure of compliance provides a snapshot of contamination, but it contains very limited information about both the degree of contamination and its health significance. Presence/absence measurement was developed for monitoring where contamination was infrequent (Pipes et al. 1987), but because of its ease of use and lower cost, it has been frequently applied where water contamination is common. When monitoring is infrequent and samples are often contaminated (on average, we found 45% of samples at the source and 71% of samples from HSW were contaminated in this meta-analysis), the data become much less useful. Although there is some evidence of a relationship between noncompliance and level of contamination (Bain et al. 2014a), a higher percentage of noncompliance does not necessarily indicate that water is more highly contaminated, but simply that contamination is widespread.

*Methodological challenges: study level*. One of the main methodological challenges of this review was how to compare water quality at different sampling points from collection to consumption. We chose to analyze water quality compliance data at two sampling points, the source and HSW. Some studies (Klasen et al. 2012; Kumpel and Nelson 2013) suggest a more nuanced schema of the different points where contamination may occur, including the point of consumption, during transport, and so on. Of particular importance is the point of consumption. HSW is not consumed directly, but is transferred to at least one other container or utensil before drinking. Thus, HSW quality data are likely to underestimate contamination of water as consumed and used in food preparation. Three studies in the qualitative synthesis sampled water from the drinking cup, a point closer to consumption. However, data were insufficient to generate pooled estimates of quality. Although we present these data as two points on the pathway from collection to consumption, these group-level data may not be connected at the level of individual samples.

In some studies, water points and households were sampled separately and data were then aggregated by water supply and matched *post hoc*. Some of these studies sampled more HSW than sources, where more than one household used a given source. In others, all sources in a study area were sampled, but HSW was only sampled for a fraction of sources. Aldana (2010) and Aliev et al. (2010), which included water supplies where noncompliance was lower in HSW than at the source, fall into this latter category. Although it is possible for quality to improve between source and storage (VanDerslice and Briscoe 1993), in both of these studies (Aldana 2010; Aliev et al. 2010) HSW was sampled for only 10% of sources and source and HSW quality were not linked. Because our analysis includes all HSW and source samples, we cannot draw inferences about individual samples.

Even for studies with a paired sampling design—with one water sample taken at a household’s source and one from their water storage container—there are several reasons these samples cannot be considered “true” pairs. Whereas households collect water and store it for a period of time, researchers often take samples closer together in time, often sampling HSW and then following up with source sampling after source identification by households.

Collecting data over a period of 2 years and visiting some households > 10 times, Trevett et al. (2004) found high variation in contamination levels of samples taken over time from the same household, which they attributed to household behaviors. At the source, variability of contamination may be caused by factors such as seasonality (Kostyla et al. 2015). This potentially high variability in contamination at both the source and in HSW combined with the temporal dislocation in sampling noted above may introduce error. In addition, significant natural attenuation has been found in indicator organisms in stored water over time (e.g. Levy et al. 2008), but longer storage time also presents more opportunities for contamination. Variation in how long water has been stored in the household before sampling is thus likely to be a source of heterogeneity in water quality data, both within and between studies, along with other factors such as temperature.

Heterogeneity of studies was very high. In addition to the methodological issues for measuring and comparing noncompliance listed above, this high level of variation reflects the fact that water sources and household water storage are located within complex systems. Some aspects of these complex systems that impact noncompliance are touched upon in the included studies. For example, studies may have reported the proportion of participants treating water, but FIB data disaggregated by household treatment were rarely reported, preventing meta-analysis of household treatment. Rural/urban geography was reported in the studies; however, population densities for what is considered rural and urban may vary widely between and even within countries (Christenson et al. 2014). Our knowledge of these complex systems is limited and this high variability, which remains after exploring known confounders and effect modifiers, is evidence that we have a lack of understanding the system in which contamination at the source and in HSW occurs.

*Methodological challenges: review level*. In their original review on source versus stored water quality, Wright et al. (2004) collapsed noncompliance data on quality at the source and quality in HSW into a single odds ratio. The associated loss of information and dimensionality inhibits analysis of which covariates are relevant at each sampling point. Bivariate techniques were developed for meta-analyses of sensitivity and specificity of medical diagnostic tests in order to avoid the loss of data and dimensionality associated with the use of the diagnostic odds ratio (Menke 2010; Reitsma et al. 2005). Assuming a bivariate normal distribution for noncompliance at the source and in HSW, we apply the same analytical technique to water quality.

While bivariate techniques are becoming more common in meta-analyses of medical diagnostic tests, they have not been frequently used outside that field. Using both bivariate and univariate methods has allowed us to avoid data and dimensionality loss of converting noncompliance at the source and in HSW to a simple odds ratio while also exploring the relative contamination at both sampling points. Testing for heterogeneity in bivariate and multivariate meta-analysis is a new but rapidly developing field; bivariate methods for exploring publication bias have not yet been developed, thus necessitating use of univariate tests. The most recent literature suggests that the most appropriate application of a univariate method to test for publication bias is to apply the trim and fill method to the log odds ratio of the two variables (Bürkner and Doebler 2014).

One assumption of this meta-analysis is that in studies that have data on multiple water supply types, the contamination of each of these supplies is independent of the others. Because water quality is affected by environmental sanitation and other community-level factors, this assumption may not always hold true. In addition, this method gives more weight to studies with a higher number of water supply types studied. However, because most of the studies explored only one supply type and only 7 of 45 studies included three or more supply types, it is not feasible to group water supplies by study for analysis.

Finally, our review was limited by the number of studies identified for some supply types and the quality of reporting. In particular, infrequent and inconsistent reporting of residual chlorine meant that we were not able to determine whether this was one of the main reasons piped supplies were less likely to be contaminated. Lack of a definition of HSW may have contributed to high heterogeneity within results for the stored water quality. In addition, it is possible that we missed studies due to lack of standard terminology. We suggest that adopting a definition of HSW will enable better comparisons across studies and contexts.

## Conclusions

We found substantive evidence for deterioration in water quality between source and stored water. Therefore, estimates of the global population drinking safe water, even those that account for water quality and sanitary inspection, are likely to be overestimates due to contamination during collection, transport, or storage. We propose that monitoring of drinking water quality should occur at both the source and in HSW. We found that piped water was significantly less likely to be contaminated than other water supply types, both at the source and in HSW, and thus suggest that a shift toward piped supplies will lead to both improved quality and safety of drinking water in the household. Although previous development policies have focused on extending a basic level of service to all, we suggest that future development policies, such as the Sustainable Development Goals, need to incorporate goals of moving people up the water ladder. HWTS may have a role to play in the short term, but improving source water quality—particularly of piped sources—is likely to lead to improved quality at both the source and in HSW. In particular, a consistent supply of high-quality piped water on the premises is likely to lead to the highest quality drinking water, even if storage continues. We see a role for the health sector in surveillance and policy making to address the interaction between stored water quality and household hygiene. To evaluate the success of future development policies in providing safe drinking water, future studies should seek to move beyond presence/absence measures to report FIB or even pathogen counts and variances in addition to recording and reporting residual chlorine using the WHO guideline values of 0.2 and 0.5 ppm (WHO 2011).

## Supplemental Material

(287 KB) PDF
