# Supplementary material for: Association of Supply Type with Fecal Contamination of Source Water and Household Stored Drinking Water in Developing Countries: A Bivariate Meta-analysis
Source: Environ Health Perspect. 2015 May 8;123(12):1222–31. doi: 10.1289/ehp.1409002 (PMC4671240; doi:10.1289/ehp.1409002)
Supplement: (287 KB) PDF [file ehp.1409002.s001.acco.pdf]

Environ Health Perspect
doi: 10.1289/ehp.1409002

Note to Readers: *EHP* strives to ensure that all journal content is accessible to all readers. However, some figures and Supplemental Material published in *EHP* articles may not conform to 508 standards due to the complexity of the information being presented. If you need assistance accessing journal content, please contact ehp508@niehs.nih.gov. Our staff will work with you to assess and meet your accessibility needs within 3 working days.

Supplemental Material

Association of Supply Type with Fecal Contamination of Source Water and Household Stored Drinking Water in Developing Countries: A Bivariate Meta-analysis

Katherine F. Shields, Robert E.S. Bain, Ryan Cronk, Jim A. Wright, and Jamie Bartram

Table of Contents

Table S1. Quality criteria used to assess studies of microbial water quality in developing countries.

Figure S1. Map of locations of studies.

References

Table S1. Quality criteria used to assess studies of microbial water quality in developing countries.

Criterion	Question
Selection randomized	Was sampling randomized over a given study area or population?
Selection described	Do the authors describe how the water sources were chosen, including how either the types of water source or their users were selected?
Region specified	Does the study report the geographic region within the country where it was conducted?
Season reported	Were the seasons or periods during which sampling and/or inspections took place reported?
Representative	Was the study designed to provide representative picture water quality in a given area?
Quality control	Were quality control procedures specified?
Method described	Are well-defined methods of analysis described or referenced?
Point of sampling	Was the point at which water was sampled well-defined? (For example whether the water was collected from within a household storage container or directly from a water source)
Handling described[a]	Are sample handling procedures described, including sample collection, method and duration of transport (if required) and incubation temperature?
Basic handling criteria[b]	Does sample handling and processing meet the following criteria: transport on ice or between 2-8°C or field-testing, analysis within six hours of collection and specified incubation temperature (35 or 37 ±1°C for EC and 44.5 ± 1°C for TTC)?
Accredited lab	Was the microbial analysis conducted in an accredited laboratory?
Trained technician	Do the authors state whether trained technicians conducted the water quality assessments?
External review	Was the study subject to peer review?

Studies were assigned a point for each item in Table S1 resulting in a study quality score of between 0 and 13, and then broken into terciles of "low"(<7), "medium" (7-9) and "high" (>9) quality.

[a] Studies referencing Standard Methods for the Examination of Water and Wastewater (Rice et al. 2012) or other national or international standards are assumed to have followed the procedures outlined in these publications, i.e. that they stipulate quality control and handling procedures.

[b] The criterion for analysis within 6 hours is more stringent that those specified in most standards, including the Standard Methods for the Examination of Water and Wastewater (Rice et al. 2012) and the International Organization for Standardization (ISO) but is generally recommended for microbial water quality analysis (WHO 2011).

Source: Bain et al. (2014)

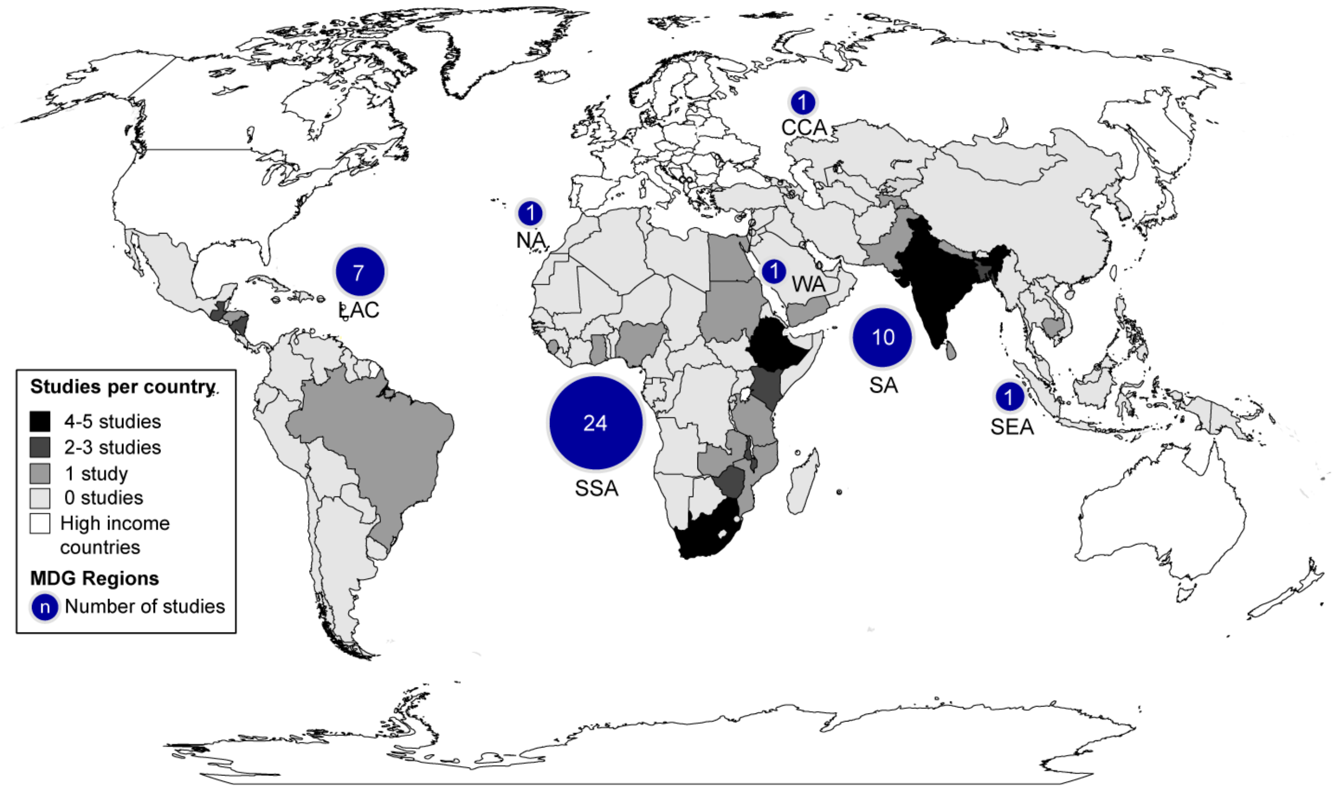

Figure S1. Map of locations of studies.

References

Bain R, Cronk R, Wright J, Yang H, Slaymaker T, Bartram J. 2014. Fecal contamination of drinking-water in low-and middle-income countries: a systematic review and meta-analysis. PLoS medicine 11(5): e1001644.

Rice EW, Bridgewater L, American Public Health A, American Water Works A, Water Environment F. 2012. Standard methods for the examination of water and wastewater. Washington, D.C.: American Public Health Association. Available: https://www.standardmethods.org/ [accessed 26 April 2015].

WHO. 2011. Guidelines for drinking-water quality. 4th ed. Geneva: World Health Organization. Available: http://whqlibdoc.who.int/publications/2011/9789241548151_eng.pdf [accessed 22 April 2015].
